# Playing a video game is more than mere procrastination

**DOI:** 10.1186/s40359-019-0309-9

**Published:** 2019-06-13

**Authors:** Kent Nordby, Ronny Andre Løkken, Gerit Pfuhl

**Affiliations:** Department of Psychology, UiT The Arctic University of Norway, N-9037 Tromsø, Norway

**Keywords:** Choice impulsivity, Computer games, Temporal discounting, Internet gaming disorder, Media use

## Abstract

**Background:**

Procrastination is seen as a severe problem among young people, and many factors have been claimed to be associated with it, playing video games being one of them. One of the reasons why video games might be related to procrastination is their ability to offer instant gratification and feedback, while at the same time offer distractions from less tempting and rewarding tasks. It is not yet agreed on whether or not video game players are more prone towards procrastination and discounting of future rewards.

**Method:**

Over 500 participants across two studies completed two surveys on video gaming habits, as well as a measurement of procrastination tendencies. In study 1 participants performed an experiential discounting task, while participants in study 2 performed the 5-trial adjusting delay discounting task, both tasks assessing preference for delayed larger rewards.

**Results:**

In study 1, hours of videogaming was not significantly related to procrastination or the discount rate. In study 2, hours of videogaming was not strongly associated with procrastination and delay discounting either. However, when asked why they play, those answering to escape reality and to reduce stress had more problems of procrastination than those who play for entertainment, reward or social reasons. Overall, the association between procrastination and hours spent playing video games was weak but positive, *r*(513) = .122.

**Discussion:**

Time spent enjoying and engaging in video gaming is done for various reasons, only for a few this is related to procrastination. By using only hypothetical payouts in the discounting tasks, the absence of a relationship between hours spent video gaming, procrastination and delayed gratification requires further investigation. However, playing video games is more than mere procrastination.

## Background

From the 70s arcade classics Pong and Space Invaders to modern day triple-A games such as Halo and Grand Theft Auto, video games have gone from being a phenomenon at the local arcade-halls to an integral part of the daily lives of millions of gamers around the world. With a multi-billion dollar industry that now far surpasses Hollywood in revenues [1], game developers around the world are fighting to find ways to attract gamers to their products and keeping them there. With video games ability to provide pleasurable experiences, be highly motivating, entertaining and immediately rewarding [2], there is a preconception that gamers run the risk of getting distracted from their less engaging real-life obligations, preferring to play games instead. The scientific literature is scarce in regards to non-pathological video-gamers, their procrastination and the effect of related reward mechanism in games. It is possible that games in combination with a preference for immediate rewards can create the “perfect storm”, with excessive gaming and procrastination as a result. However, not everything is negative, as the use of games in teaching and learning is steadily growing, utilizing some of the same mechanics seen in purely recreational games (e.g. Crystals of Kaydor, [3]). We here present two studies on video gaming, procrastination and delay discounting. First, we briefly review factors contributing to procrastination, and ways to procrastinate illustrated on media consumption.

### Procrastination and media usage

Procrastination, aka “voluntarily delay an intended course of action despite expecting to be worse off for the delay” [4], has seen a rise in popularity both inside and outside the research community in recent decades. Being described as the quintessential form of self-regulation failure [4], the core of procrastination is consistently shown to be a result of self-regulation failure in both quantitative and qualitative articles [5]. It should be noted that not all self-regulation failure is procrastination (i.e. getting stupendously drunk might be a result of self-regulation failure, but it is not procrastination), but all procrastination is a result of self-regulation failure in one form or another. Procrastination has been shown to reduce with age and affects both genders equally [6], and approximately 15–20% of the general population struggle with problems of procrastination [7, 8]. While some forms of delay is normal and acceptable, habitual or chronic delay is characterized by the irrational choice to delay despite knowing that it will result in negative consequences [4]. Problems of procrastination have been associated with several negative effects to both physical and mental well-being [4, 9]. Those struggling with procrastination experience higher levels of anxiety and depression, worry [10], feelings of guilt [11], as well as increased stress and reduced well-being [12]. In addition, studies show that procrastinators also neglect their physical well-being, often delaying going to necessary physical exams, doctors’ and dentist appointments [9, 13, 14], as well as performing less wellness behaviors such as healthy eating and exercising [14].

While a large body of research on procrastination has investigated the relations between personality traits and procrastination [15], impulsivity has been received extra attention due to being one of the strongest correlates of procrastination [16]. Several findings imply a connection between impulsivity and procrastination [4, 5, 17], with higher impulsivity being related to more procrastination. However, impulsivity is not a unitary construct [18] and experimental tasks measure different aspects of impulsivity [19]. One well-established paradigm to gauge impulsivity is delay discounting, i.e. the extent to which smaller and immediate rewards are preferred over larger and delayed rewards [20, 21]. Such a paradigm has been used in a recent study [22], with results showing that procrastinators had a higher preference for immediate rewards compared to non-procrastinators. These findings are in-line with other research indicating that procrastinators have a higher tendency to engage in short-term mood repair when faced with a task that is viewed as aversive [13], as well as a lower ability to delay gratification [23]. One way that procrastinators can find their short-term mood repair and escape from the chores of everyday life is through the use of various forms of media. For those who are well regulated, media can be a source of relaxation and recovery from the strain of daily life [12, 24]. For others, media can be a form of psychological escapism, with the wish to escape from ruminating on negative events or unsolved problems in their lives [25]. Although correlational, individuals who report lower life satisfaction and well-being have been found to watch more television than individuals with less stress and those who reported a higher quality of life [26, 27]. Indeed, a growing number of publications indicate that increased media use is also linked to problems of procrastination [28–30], where media consumption can result in exacerbating problems rather than alleviating them. In a recent study among students, those reporting low trait self-control, also reported more habitual checking and enjoyment of Facebook, suggesting that Facebook can be a tool for procrastination [31]. Similarly, it has been found that low trait self-control was related to increased time spent on leisure media use and decreased time on self-directed learning [29]. It seems then, that those who procrastinate frequently, use easily accessible entertainment such as TV, internet and video games to escape from their more important obligations [32, 33].

### Advantages and disadvantages of playing video games

With games becoming more widespread and readily available [34], games can now serve as a medium for procrastination alongside television and the internet [32]. Previous research has demonstrated that those who chronically delay (i.e. procrastinators) have a high preference for pleasurable activities such as games as distractors from aversive tasks [11]. This aligns with an experimental study [28], showing that reducing internet gaming can help reduce procrastination and increase life satisfaction. Some studies have also found that too much video gaming is related to negative effects such as lower psychosocial well-being and loneliness, poorer social skills, decreased academic achievement, increased inattention and decreases in verbal memory performance [35], but these findings remain mixed and controversial [36]. As such, more recent research has shown that these negative effects of video game play is not ubiquitous, with newer studies have started documenting that playing video games can also have several positive effects. For example, in a meta-analysis [37] playing action computer games were found to positively affect spatial skills and that these training effects could transfer to other spatial tasks outside the video game context (but see [38]). Other positive effects of video games include higher attention allocation [39], enhanced creativity and problem-solving skills [40], as well as increase in positive emotions, promote relaxation and ward off anxiety [41]. Some researchers have pointed out the important recreational value of interactive media such as games in assisting in the necessary recuperation from daily stress, and that this can lead to increased productivity in the long run [42]. Other research points out that the connection between video games and procrastination only exists when gaming is in the clinical spectrum [43], supporting the idea that playing video games can be used as an escape from problematic real life situations rather than being the source of them [44]. Importantly, there is a huge variety of video games, ranging from action / adventure games to strategy games and (social) multiplayer games that affect those who play them in different ways. Gaming has also become a popular sport with professional players, i.e. gaming has become a full-time job for a few. Video gamers are everything but a homogenous group.

To investigate to what degree video gaming is a medium for procrastination, we conducted two studies. In study 1, we explored the connection between gaming hours and procrastination in a Norwegian sample. We also measured sensitivity to delay discounting with hypothetical rewards. Our rational was that highly impulsive individuals should have more problems resisting the “pull” of easily accessible and entertaining games, resulting in more time playing games, and more delay doing other important tasks, i.e. procrastination. In study 2, an online survey, we asked also for the reasons of playing video games, as this can be an important factor for whether or not gaming is a sign of procrastination, or just a relaxing pastime. In this survey we also used two short discounting tasks, one temporal and one effort discounting task. Our rational was that those procrastinating using video games also display cognitive and temporal discounting.

## Study 1: video gaming, procrastination and experiential discounting

If procrastinators are more likely to play video games, and have a stronger need for immediate reward, then one would expect that many hours of video gaming and a high degree of delay discounting is common among procrastinators. That is, we expect that the more one is engaging in activities that provide immediate reward such as video gaming the more one procrastinates. Note that we did not focus on internet gaming, but asked for engaging in any computerized game, offline and online.

## Methods

### Participants

A total of 663 participants were recruited through social media (Facebook, reddit), e-mail and bulletin boards at various Universities in Norway (28.2.-7.3.2016). Survey language was Norwegian. Of those, 393 finished the questionnaire (286 male, 72.8%); and 119 (85 male, 71.4%) finished both the questionnaire and took the experiential discounting task (EDT). Participants were between 18 and 60 years (*M* = 25.6, *SD* = 6.8). The low completion rate (17.9%) can be explained by participants having to install the Inquisit Web Player (Millisecond.com) for performing the EDT. Furthermore, of the 119 only 102 had valid EDT results, e.g. completing all rounds.

### Materials

#### Experiential discounting task (EDT)

Sensitivity to delay discounting was assessed with the Experiential Discounting Task [45]. The participant makes choices between a standard amount (3 NOK) that was probabilistic (35% chance of receiving) and delayed (0, 7, or 14 s) and an immediately guaranteed reward that was adjustable (starting at 1.5 NOK). The adjustable sum increased in the next round if the fixed sum (3 NOK) was selected, and decreased in the next round if the adjustable sum (1.5 NOK) was selected. The waiting time between each round was set to 30 s. Compared to the original task design, we adapted the currencies and used only three rounds, not four, as well as shortening the intertrial interval from 60 to 30 s.

Each round ends when the participant’s “Indifference Point” (IP) has been determined or 5 min elapsed. IP refers to the point where the subjective value of both presented sums is (apparently) identical to the participant. The IP was based on the last six choices, i.e. the average adjusting-option amount. A potential waiting time was added between each trial and after the last trial. Participants were not paid their earned winnings, but were instructed to act as if payment would occur through task instructions. By using only hypothetical rewards, [46, 47] found that the choices made in a smartphone game in over 1000 participants resembled those found in laboratory experiments using real money [48]. Similarly, [49] found no difference between hypothetical and real reward. Furthermore, [50] found that the majority of their participants were less or equally risk averse in the hypothetical compared to the real payoff conditions; but overall insensitive to the magnitude of the reward, i.e. equally risk averse whether the lottery was e.g. $1, $10 or $100. Indeed, the review by [51] found support for laboratory tasks relating to real behavior but warrant further research as e.g. the hedging problem is still not addressed fully.

#### Pure procrastination scale (PPS)

The PPS consists of 12 items [52], rated on a 5-point Likert scale (1–5) with higher scores indicating more procrastination. The Norwegian version was translated and validated by [53], with the present study using a selection of 5 items from the PPS that have shown very good psychometric properties compared to alternative procrastination scales [54]. In the survey (*N* = 393) the PPS had a Cronbach’s alpha of .92, while for the sample with valid EDT results (*N* = 102), PPS had a Cronbach’s alpha of .928 (95% CI: [.903; .948].

#### Video game usage and history

Five questions were used to address the participants’ video game usage and history. Participants were asked how many days they spent gaming each week, hours per day, type of video game (action, adventure, role-playing games (RPG), simulation, sport, strategy), device used (PC, console, mobile phone) and age started video gaming. Type of video game was rated on a 5-point Likert scale from 1 – playing this type not at all/rarely to 5 = playing this type of game very often; device used was measured as a percentage.

### Procedure

The study was online and took about 15 min to complete. Participants read first a short description of the purpose of the study, contact information, and by proceeding gave informed consent. They were then presented with questions on their video game usage and history, and answered the 5-item PPS. The survey was implemented in Qualtrics (Qualtrics.com). At the end of the questionnaire, each participant was asked to proceed to download the Inquisit Web Player (Millisecond.com, 3.1 MB large) in order to perform the EDT task.

### Analysis

The number of hours played video games was the product of the number of days per week and hours per day. We excluded data which indicated video gaming for over 100 h, i.e. more than 14 h on all 7 days (*N* = 2). The procrastination score was the average score from the five PPS items. We followed the procedure of [45] to calculate the exponential discounting value k, where a higher value of k equals higher discounting (i.e. that the participant want a higher reward for delay). For 17 participants we could not calculate a (sensible) k value as they had no valid value in at least one of the three rounds (*N* = 10 in round A, *N* = 1 in round B), a negative value in one round (*N* = 2), and four participants showed the reverse of discounting. Thus, the analysis for the EDT is based on 102 participants. Note, due to using only three rounds instead of four rounds, some non-linearity / non-monotonic performance was found too, as well as two participants had no discounting at all, but this led not to exclusion from data analysis.

The individual k-values (*N* = 102) and hours video gaming were predictors with the PPS score as outcome. We also run a regression model were we additionally controlled for age and gender [6]. Data analysis was done in JASP [55].

## Results

Of the 393 participants that finished the survey 30% took the EDT. There was no difference in age, gender, amount of video gaming, video gaming experience, type of games mostly played or device used for gaming among those that finished the survey only and those that took the survey and proceeded playing the EDT (Table 1). Approximately 12% of participants did not engage in video gaming.

**Table 1:** Demographics of the sample and the subsample completing both parts of study 1

	Survey only (*N* = 393)	Survey+EDT (*N* = 119)	Survey + k_EDT (*N* = 102)
age, *M* ± *SD*	25.47 ± 6.67	25.46 ± 6.32	25.64 ± 6.27
Male; Female (% male)	286; 107 (73%)	85; 34 (71%)	75; 27 (74%)
# players; non-players	342; 41	105; 14	90; 12
PPS score, *M* ± *SD*	2.98 ± 1.05	3.17 ± 1.1	3.15 ± 1.08
hours of video gaming	16.36 ± 16.2	15.91 ± 14.07	16.09 ± 14.7
years of video gaming	10.39 ± 5.27	10.51 ± 5.85	10.4 ± 6.0
PC; console; mobile	64;23;13	64;23;13	67;19;14
Action / Adventure	3.05 ± 1.35	3.09 ± 1.32	3.04 ± 1.35
RPG^a^	3.20 ± 1.49	3.35 ± 1.49	3.29 ± 1.52
Simulator	2.14 ± 1.23	2.25 ± 1.24	2.3 ± 1.3
Strategy	3.31 ± 1.25	3.19 ± 1.32	3.29 ± 1.27
Sport	1.88 ± 1.28	1.73 ± 1.17	1.76 ± 1.18

^a^Role Playing Game

Most people played strategy, RPG or action and adventure games. Sport and simulator games were the least played type of video games. Women played equally on PC, console or mobile phone whereas men played nearly four times more on PCs (χ^2^_393_ = 89.215, *P* < .001) than on console and mobile phones.^1^

Among the 102 participants where the discounting rate could be estimated the null model of predicting procrastination severity from video gaming hours and sensitivity to discounting (k value) was not statistically significant, *F*(2, 101) = 3.040, *P* = .052, *R*^2^ = .058. The model including age and gender was statistically significant, *F*(4, 101) = 3.012, *P* = .022, *R*^2^ = .11, where age: β = −.241, *P* = .019, and hours of video gaming: β = .219, *P* = .030 but not discounting rate (*P* = .119) or gender (*P* = .362) related to PPS. That is, the older the participant the less procrastination, and the more hours spent video-gaming the more procrastination. Crucially, we did not find that delay discounting related to procrastination, *r*(101) = .153, *P* = .124. The correlation between PPS score and video gaming hours in the survey only sample was *r*(392) = .068, *P* = .181. Older participants played fewer hours of video games, *r*(392) = −.151, *P* = .003, and also had a lower PPS score *r*(392) = −.115, *P* = .023.

## Discussion

The purpose of study 1 was to investigate if more hours of video gaming and stronger delay discounting could predict more problems of procrastination. The results showed no strong support. We did not find that more delay discounting in combination with more hours spent on video games predicted more problems of procrastination. We did not find that delay discounting was related to procrastination either. Further, although there was an association between hours played video games and procrastination, this link was weak and only in an analysis taking age and gender into account. As previously reported, procrastination was less the older the participant was [6]. With age also the number of hours spent video gaming declined. Likely, as one gets older other obligations, i.e. family and job, or not being a student, offers less time to indulge in procrastination [56]. The experiential discounting task might also appeal to procrastinators, as the waiting time could be used to e.g. check something on the smartphone. That is, the survey and playing the discounting task are itself means to procrastinate.

Since not all video gamers are students or teenagers, our study is more generalizable, despite being a convenience sample, than a study done solely on a student population. Furthermore, despite a large amount of dropouts our results were unlikely affected by selection bias (Table 1), as we found no systematic differences between those that choose to complete the EDT plus the questionnaire versus those that completed the survey only. Perhaps contrary to popular belief then, the final result showed that increased amount of gaming hours had only a small impact on procrastination, and was not modified by delay discounting, i.e. the degree that someone prefers smaller immediate rewards as opposed to larger but delayed rewards. Indeed, [57] found no relationship between hours playing video games and negative outcomes, suggesting that measuring video game hours alone is insufficient.

One potential problem in generalizing results from this study is that Norwegian youth report a lower prevalence rates of gaming addiction compared to some other countries. While only 0.9% of Norwegian youth reach criteria for gaming addiction [58], other countries report a much higher prevalence such as the United States (8.5%; [59], Singapore (8.7%; [60], Netherlands (1.9–2.3%; [61] and South Korea (2.7%; [62]. However, true prevalence rates for internet gaming disorder might be between 0.3 and 1.0%, as found in four international cohorts [63], somewhat higher among younger adults than older adults but in all four cohorts it had a lower prevalence than pathological gambling. Furthermore, [64] recommended to be more cautious about diagnosing someone with a gaming disorder, as it is not yet clear whether internet gaming disorder may just be a subcategory of internet addiction disorder or any other behavioral addiction.

In relation to procrastination, worth noting is that one of the criteria for gaming addiction is to answer positive to “neglect other important activities (e.g. school, work, sports) to play games” [61], which was among the questions reported as least problematic in the Norwegian report [58].

In relation to the experiential discounting task, [65] have criticized the validity and construction validity of the task, and claim that the experiment probably measures something else. However, [66] found that the task has strong reliability and validity and recommend it for measuring choice impulsivity in humans.

## Study 2: video gaming, procrastination, delay and effort discounting

Study 1 yielded no strong association between time spent playing video games and procrastination, nor was there any association between discounting and procrastination. It is possible that the latter might be due to the experiential discounting task in study 1, as the probabilistic component of the task may appeal to some gamers. Procrastination has mostly been linked to delay discounting [4, 22]. In a sample of Chinese students (*N* = 47) [22] found a large effect size between low and high procrastinators. However, one may discount due to having to wait for the (bigger) reward, due to it being less than certain to receive the reward, or due to it being too effortful to receive the reward [67]. Because task aversion is related to procrastination [68], and having to spent more effort is related to task aversion [69], it is plausible that procrastination relates to effort discounting and avoiding cognitively demanding tasks, respectively [69, 70]. Accordingly, in the current study we expected a link between procrastination and video-gaming if the reason for playing video games is for task aversion, i.e. escapism, break from daily activities and stress relieve. Further, procrastination might be predicted by a preference for immediate reward and easy tasks. We used a short delay discounting task and explored effort discounting by using a very short beads counting task [71]. Finally, we recruited an international sample as video gaming is not so prevalent in the Norwegian population.

## Methods

### Participants

A total of 171 participants were recruited through social media using English websites, and bulletin boards at UiT The Arctic University of Norway (19.2.-4.3.2018). Of those, 123 finished the questionnaire (72 male, 59%), of which 82 took the English survey and 41 the Norwegian version of the survey. Participants were between 16 and 59 years old (M = 29.1, SD = 9.2), three participants did not disclose their age.

### Materials

#### 5-Trial adjusting delay task

Developed to quickly obtain a discounting rate [72], this task assesses the discount rate k by using a stair-case procedure where the delay to the larger amount is adjusted to determine the effective delay 50% (ED_50_). The ED_50_ values were on a logarithmic scale. The first choice trial was between 1000 NOK (or $100 in the English version) delayed 3 weeks and 500 NOK ($50) available immediately. In the next trial, the delay either adjusts down (immediate choice) or up (delayed choice) by 8 delays on the logarithmic scale (see Table 1 in [72].

#### Effort discounting task

In this task, participants were presented with a matrix consisting of an unequal number of blue and red beads, where they had to indicate the color of the majority of the beads [71]. There were 5 trials, showing in each trial 100 beads in a 10 × 10 matrix. The first trial had 45 blue beads, with the remaining having 49, 48, 51 and 47 blue beads respectively. We recorded the time spent on the page, as a measure of how long it took the participant to solve this item. We reasoned that guessing is faster than counting and given the low number of trials used, guessing five times correctly was possible in 3.1% (1/2 ^5).

#### Pure procrastination scale (PPS)

Procrastination was evaluated using the 5-item version of the PPS as in study 1. In study 2 Cronbach’s α of the scale was .92, 95% CI [.89; .94].

#### Video game usage, history and purpose

Video gaming hours was assessed similarly to study 1, i.e. we asked for how many days they spent gaming each week, hours per day, type of video game (action, adventure, offline role-playing games, online massive role playing games, simulation, sport, strategy / multiplayer online battle arena (MOBA)), device used (PC, console, mobile phone) and age started video gaming. In addition, participants were asked why they played video games, offering seven answer options: entertainment, escape from reality, competition/training, social gathering, break in everyday life, break from stress, or for reward. Multiple answers were permissible. After selecting their responses, participants had to rank these reasons by importance. We also asked whether they play professionally and or have programmed / developed (parts of) video games.

### Procedure

The experiment was online and took about 8 min to complete. Participants read first a short description of the purpose of the study, contact information, and by proceeding gave informed consent. They were then presented with questions on their video game usage and history, interleaved with the 5-item PPS, the 5-trial adjustable delay discounting task, and the effort discounting task. Lastly, we asked for when they started to play video games, their age and gender. The survey was implemented in Qualtrics (Qualtrics.com).

### Analysis

As for study 1 we calculated hours of video-gaming as the product of days played and hours per day played.

The individual discount rate (k-values) was calculated using the procedure used by [72]. We excluded one participant who was willing to wait for 25 years.^2^ For the effort discounting score, we calculated the number of times the participant correctly selected the majority color. Since errors are most likely due to not having counted the beads, we calculated the (average) response time and we correlated these response times of errors (51 participants had at least one error) with the procrastination score. We treat this analysis with caution as response times in Qualtrics depend on many factors, e.g. differences in speed of internet connections, or unforeseen interruptions. Significance level was adjusted for multiple comparisons where appropriate, e.g. for device usage: α < .017, for type of game: α < .008. We expected at least a medium effect size (based on the large effect size reported in [22], and a sample of *N* = 82 would have had a power of .8 to find an effect, i.e. bivariate correlation of .3 between delay discounting and procrastination (G power 3.1, [73]).

Data analysis was done in JASP [55].

## Results

Of the 123 participants who completed the survey, 37 participants indicated that they do or have done programming / development of video games, and 7 participants said they play professionally. Ten participants did not play video games. There was no difference between those who developed games and non-developers in the number of hours played: t(121) = 1.582, *P* = .116, *d* = .311, in the PPS score: t(121) = .871, *P* = .385, *d* = .171, or their discount rate^3^: t(119) = .997, *P* = .321, *d* = .198, but there was a difference in the effort discounting score with programmers having on average a score of 4.7 (*SD* = .525) whereas players only had an average number of correct answers of 4.4 (*SD* = .761), Welch’s t (93.794) = 2.106, *P* = .038, *d* = .386. Next, we looked at differences between the English and Norwegian respondents, with details provided in Table 2. The Norwegian respondents indicated lower hours of video gaming than the international sample. This effect remained even when excluding the non-playing respondents (*P* = .024).

**Table 2:** Comparison of the Norwegian and English survey respondents

	Norwegian respondents (*N* = 41)	English respondents (*N* = 82)	
age, *M* ± *SD*	29.03 ± 9.27	29.26 ± 9.2	*P* = .898
Male; female (% male)	20, 19 (51%)	52; 30 (63%)	*P* = .088
# players; no players	36; 5	77; 5	*P* = .004*
PPS score, *M* ± *SD*	2.62 ± 0.8	3.47 ± 1.13	*P* < .001
hours of videogaming, *M* ± *SD*	15.57 ± 17.39	26.59 ± 24.11	*P* = .010
years of videogaming, *M* ± *SD*	18.67 ± 7.72	19.31 ± 7.56	*P* = .666
Action / adventure	3.34 ± 1.34	3.11 ± 1.49	*P =* .354
Offline role playing	2.66 ± 1.25	2.14 ± 1.25	*P =* .242
Online massive role playing	2.48 ± 1.53	1.86 ± 1.36	*P* = .025
Simulators	2.2 ± 1.32	1.86 ± 1.31	*P =* .498
Strategy / MOBA	2.78 ± 1.4	2.78 ± 1.51	*P =* .87
Sport	1.39 ± .86	1.42 ± .84	*P* = .208
PC; console; mobil	64;15;20	48;20;32	All *P’s* > .05
Delay discounting k, *M* ± *SD*	.017 ± .034	.077 ± .38	*P* = .328
Effort score, *M* ± *SD*	4.4 ± .84	4.57 ± .63	*P* = .205

*using the number of days from 0 to 7 instead of dichotomizing

There was no difference in the number of those programming / developing video-games, i.e. 10 programmers took the Norwegian survey and 27 the English survey, χ^2^ = .947, *P* = .330. There was no difference in device usage between English and Norwegian survey versions, with most participants playing on the PC (59%), 17% used a console, and 24% used mainly the mobile phone. However, the international sample played more online massive role playing games than the Norwegian participants, χ^2^ = 11.185, *P* = .025, but given six categories, this does not survive correction for multiple testing. Furthermore, for the device used, we found that women played more than men on the mobile phone (*P* < .001), and men played more than women on the PC (*P* = .006), both played equally on the console (*P* = .528).

**Fig. 1:**
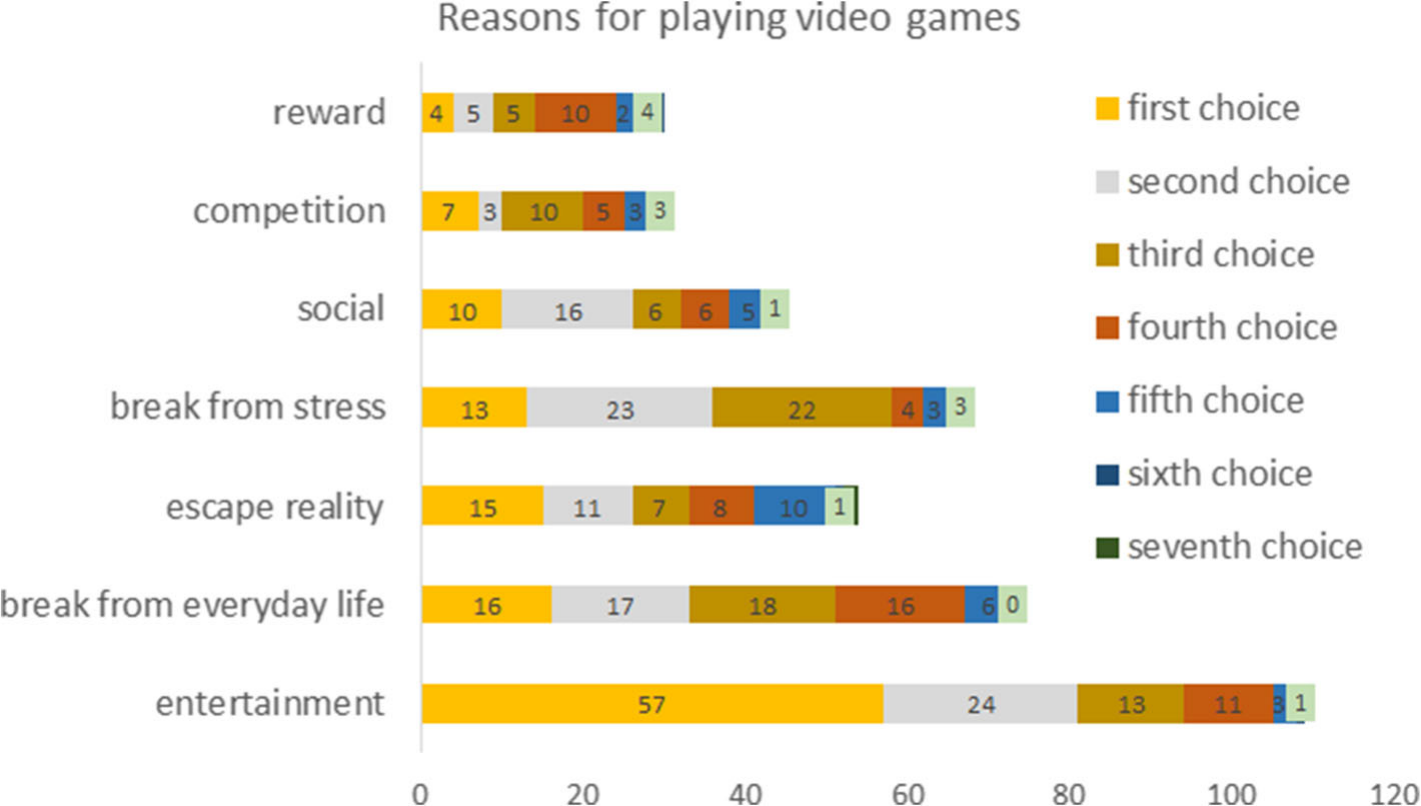
Entertainment was the most common reason, many also mentioned break from stress as first or second reason for engaging in video gaming. Less than half of the participants mentioned reward as a reason

Finally, most participants indicated playing for entertainment, and only few ranked reward and competition as first or second reason (Fig. 1).

Since we were mainly interested in the relationship between procrastination, delay discounting and video gaming, we did not include survey language as an independent variable.

Procrastination was positively but not statistically significantly associated with hours of video gaming, *r*(122) = .128, *P* = .157. This remains when considering only those playing video games, *r*(112) = .144, *P* = .128. Furthermore, procrastination was also not predicted by hours of video-gaming and delay discounting, explained variance was 4%, i.e. *F*(2, 118) = 2.461, *P =* .090. A multiple linear regression with age, gender, effort discounting, delay discounting and hours video gaming, did also not predict the PPS score, *R*^2^ = .057, *F*(5, 112) = 1.354, *P* = .247 (Table 3).

**Table 3:** Coefficients of the multiple linear regression predicting PPS score

	Unstandardized	Standard Error	Standardized	t	*p*	2.5%	97.5%
(Intercept)	2.714	0.878		3.090	0.003	0.974	4.454
Age in years	0.012	0.012	0.100	1.036	0.302	−0.011	0.035
Gender	−0.046	0.210	−0.020	−0.219	0.827	−0.462	0.370
Effort_discounting score	−0.020	0.145	−0.013	−0.135	0.893	−0.308	0.268
k (delay discounting)	0.269	0.332	0.077	0.808	0.421	−0.390	0.927
Videogaming_hours	0.011	0.005	0.213	2.276	0.025	0.001	0.021

**Fig. 2:**
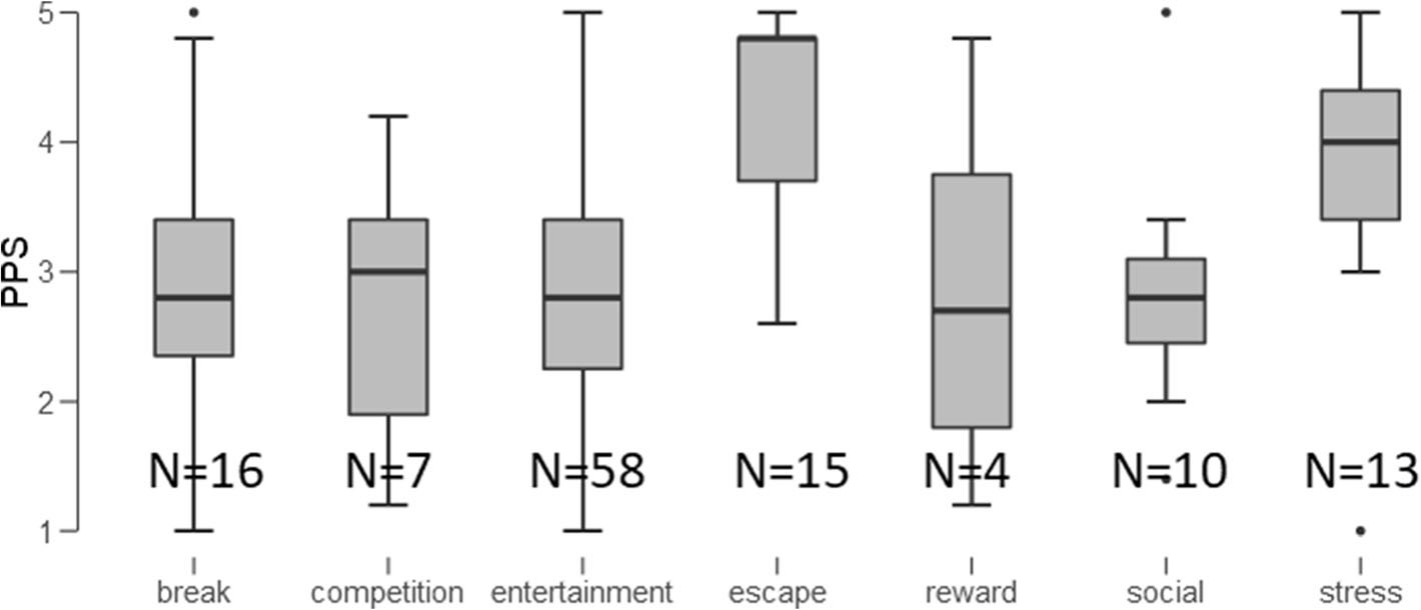
Boxplot showing the main reason of video gaming and its relation to procrastination. There was a statistically significant difference with higher PPS scores for those who indicated playing games to escape reality or for stress reduction. Tukey’s post-hoc test revealed that “escape from reality” was different from all but the “stress” and “reward” respondents (break from everyday life vs escape: t = − 3.48, *P* = .011, *d* = − 1.314; competition vs escape: t = − 3.264, *P* = .022, *d* = − 1.608; entertainment vs escape: t = − 4.296, *P* = < .001, *d* = − 1.3; social vs escape: t = − 3.358, *P* = .016, *d* = 1.532)

Next we performed an ANOVA. There was a significant difference in PPS depending on the reason why they played video games, *F*(6, 116) = 4.645, *P* < .001, η^2^ = .194 (Fig. 2). Post-hoc Tukey tests showed that playing to escape differed from break (*P* = .011), from competition (*P* = .022), from entertainment (*P* < .001), and from social (*P* = .017). However, the reason why they played, did not affect the number of hours played, *F*(6, 116) = .805, *P* = .568, η^2^ = .040. Reason of playing did also not relate to effort discounting, *F*(6, 115) = 1.7, *P* = .127, η^2^ = .081 or delay discounting, *F*(6, 114) = .958, *P* = .457, η^2^ = .048.

There was a negative correlation between PPS and the average response times of erroneous trials, *r*(51) = −.349, *P* = .016, i.e. the faster a person did the effort discounting task the higher the PPS score. This is preliminary and requires further investigation into the relationship between effort discounting and procrastination.

### Combining study 1 and 2

Combining the data from study 1 and study 2 into one statistical analysis, we found a weak but statistically significant positive correlation between the number of hours played video games and the PPS score, *r*(515) = .122, *P* = .005, 95% CI [.036; .207]. A linear regression with age and video gaming hours as predictors explained 1.8% of the PPS score, *F*(2, 511) = 4.766, *P* = .009, with video gaming (β = .127, *P* = .004) but not age (β = −.037, *P* = .405) statistically significantly contributing.

Next, we also looked whether delay discounting would be related to procrastination. In an ANCOVA with study 1 and 2 as between subject factor (as we used two different discounting paradigms) yielded no significant main effect for study, *F*(1, 219) = .333, *P* = .564, η^2^ = .001, or the discounting factor k: *F*(1, 219) = .758, *P* = .385, η^2^ = .003 but the co-variate video gaming hours was statistically significant: *F*(1, 219) = 6.67, *P* = .01, η^2^ = .029. Neither in study 1 nor in study 2, did we find a medium effect size of delay discounting and procrastination.

## Discussion

Video games are played for various reasons, although the majority play video games for entertainment purposes, some choose it as a break from daily activities, escapism or stress reduction.

Our data did not support any strong relationship between hours of videogaming, procrastination, and delay discounting, and effort discounting. In both surveys we found no statistically significant relation between hours spent video gaming and procrastination, nor between delay discounting and procrastination. The associations had a small effect size but were all in the predicted direction. Only in combining the data from both studies could we find a very small, but statistically significant, relation between procrastination and time spent on video games. We caution this result, as we advertised the study as being about videogaming, likely leading to a collider bias i.e. we may have recruited those gamers who are more prone to procrastinate than would be found in the population of all gamers. For example, recent surveys on internet gaming found a low prevalence [63] of problematic gaming, i.e. playing as escapism might be rarer in the population than in our sample.

Indeed, as expected we found that those who indicated that they were playing video games as a mean to escape from reality, or to have a break from stress, had a significantly higher level of procrastination than those who were playing for entertainment, break from everyday life, or for the social value of games. Curiously, even though it seems that these participants were using video games to procrastinate, they did not report more time playing video games than those who were procrastinating less. This strengthens previous findings that hours of video gaming is not related to severity or negative outcomes [57, 63], but that the reasons for playing video games does.

In this study 2 the English sample spent more hours video gaming than the Norwegian sample. It is possible that some Norwegian respondents took the English version, and we would not expect differences due to the language in which the survey was taken [74], but this cultural difference could bias the effect size of the relationship between procrastination and video gaming. For example, [56] found that the PPS score was 2.88 in Norway, but in our study 2 it was only 2.62, which was also lower than for study 1 (PPS score: 2.98). Clearly, these comparatively small sample sizes come with uncertainty, and a margin of error that warrants caution [75].

Results from the effort discounting task showed that of those making errors, those who spent more time, which we treated as proxy for having spent more effort to correctly identify the majority color, had less problems of procrastination. This data is preliminary as a more controlled effort discounting task is required to assess whether procrastination is a general strategy to avoid demanding tasks, or specific to the task that one is postponing. We have chosen this task to avoid a high drop-out rate (compare study 1 vs study 2) but the number of trials are clearly insufficient to draw firm conclusions. However, this preliminary data is in line with the literature that more effortful tasks, and a focus on getting done quickly, is related to more procrastination [68, 76]. Future studies should measure the effort spent video gaming not just in hours but in levels of competencies achieved.

As in study 1, we did not find that the discount rate was related to procrastination, contrary to the conclusions of [22]. One possible reason for this is that video gamers respond less well to monetary rewards in discounting tasks. However, we find it unlikely that the results are due to the task chosen, as our results agree well with [72]. Furthermore, Wu et al. dichotomized their sample into low and high procrastinators, which we did not. Therefore, their large effect sizes might be highly inflated. They also used only students, a group more prone to procrastinate [56]. We therefore would like other independent replications using experimental delay and effort discounting tasks (instead of questionnaires) and measuring procrastination. Admittedly, our study was a convenience sample but we did not solely recruit students. We further took great care to ensure high completion rate by designing a short survey, and having the delay discounting task and effort discounting task followed by video game related questions. This way, most that started the survey, also completed it.

## General discussion

The purpose of these studies was to examine whether procrastination was related to hours spend video gaming and discounting. Our results indicate no strong relationship between delay discounting, hours spent on video games, and procrastination. Study 2 suggests that not delay but effort discounting might contribute to procrastination. Our data further does not support a strong relationship between video gaming hours and procrastination, but procrastinators indicate that they play for the sake of escaping from reality and to get a break from stress [25].

Our findings are thus in line with newer research showing that amount of time spent on gaming is not necessarily related to negative consequences such as procrastination. For example, [77] found that harmonious passion (i.e. when an activity is in harmony with other aspects of the person’s life) for video games was related to amount of time spent playing games, while obsessive passion (an uncontrollable urge to engage in the activity that creates intra- and interpersonal conflicts) was not. In this perspective, gamers and time spent on video games is viewed as a pleasurable leisure-time activity with the purpose of relaxing and recovering from daily stress, rather than a temporary escape from real-life obligations. In fact, several articles highlight games ability to promote relaxation and ward off anxiety [2, 41, 78], or reduce rumination [79], as well as refuting popular stereotypes that gamers are lazy, overweight, unathletic and socially inept ([63, 80]. In relation to procrastination, a non-exhaustive literature search (PsycInfo, Web of Science, Pubmed) with the terms computer gam* or video gam* and procrastinat* yielded no study that looked at the hours played video games and procrastination. Yet, there seems to exist a stereotype of gamers being lazy “couch potatoes” that care for little else than playing games [80]. It would seem then, from ours and others results, that gamers have an undeservingly bad reputation, at least when it comes to their ability to get their intended tasks done. However, it should be noted that our study is a convenience sample of gamers, and is not a representation of people who have a problematic relationship with games.

Furthermore, both playing video games and procrastinating might be merely symptomatic of other causes, e.g. depression, anxiety [64]; and people may play games to cope with other mental health issues.

### Limitations

Firstly, we relied on subjective measures and did not observe hours spent video-gaming. Using smartphone apps [46] would provide more accurate time stamps. Secondly, to assess discounting we relied on short tasks without providing monetary outcomes. These discounting tasks are usually carried out in a laboratory, and the collected prize is paid [45]. In our study, the tasks were conducted on the Internet, and the collected winnings were not paid. This may or may not reduce the validity of the results. With respect to using only hypothetical reward, a range of studies found no difference between real and hypothetical rewards for both delay and probability discounting [46, 47, 49, 81], but using hypothetical rewards may underestimate true risk aversion [50]. A second problem with using EDT or 5-trail delay adjusting task as a measure of delay discounting in gamers, is that money acts as a more rewarding reinforcer for individuals with gambling problems [82]. It is then possible that delayed monetary reward tasks are a poor instrument for measuring impulsivity in gamers, who might be more interested in the rewards that playing video games provides them with.

Thirdly, we did not concomitantly measure depressive symptoms or general well-being, nor included measures on Internet Gaming Disorder.

Fourthly, our surveys did not cover all possible factors shown to influence procrastination, e.g. we did not ask whether respondents were students or employees, or unemployed [56].

Fifthly, study 2 was statistically insensitive for small to medium effects, and even when pooling study 1 and 2, small effect sizes (*r* = .1) could not be found with 95% power. The sample size rationale was based on [22] but we did not correct the reported effect size for publication bias or uncertainty [75], but used a too simple regression to the mean approach. Furthermore, we did assume that the relationship between delay discounting, procrastination and video gaming is of similar size but without having an a priori model, it is not obvious which factor might be a moderator, or whether all three contribute to a common, unmeasured, construct. However, our main goal was to investigate whether video gamers are procrastinators and we would deem this only supportive if there would have been at least a medium effect size.

The small effect size between hours playing video games and procrastination severity reported here, needs further investigation. Our pooled data had enough power to detect a small effect size with 90% power. But we did not control for cultural effects and prevalence rates of internet gaming disorder do differ between countries [63]. However, we did neither recruit nor measure gaming disorder but only video gaming more general.

It is possible that if one uses more objective measures of actual hours played excessive gaming may be stronger related to pathological procrastination than found here. On the other hand, by using other scales to measure procrastination, and over a wider age range, there might be no relationship between procrastination and videogaming, as videogaming is just one of many means to procrastinate (e.g. [33], playing video games is just one of the play activities in adolescence, and procrastination itself is age and context-dependent [56].

## Conclusion

To our knowledge this is the first study measuring concomitantly procrastination, video gaming habits, and preference for immediate reward. We found no strong support that procrastination is linked to hours of video gaming. By using only hypothetical reward, we also found no association between delay discounting and procrastination. Potentially what and why someone plays video games not the number of hours are a more promising avenue for procrastination research.

## Data Availability

Data (de-identified) can be found here: https://osf.io/63fae/

## Abbreviations

EDT: Experiential discounting task
IP: Indifference point
MOBA: Multiplayer online battle arena
PPS: Pure Procrastination Scale
RPG: Role playing games

## Footnotes

1 The conclusion is the same if three independent t-tests are performed on the percentage each device is used and adjusted *P*-value used.

2 Results do not change significantly if this outlier remains in all data analysis

3 One participant accepted to wait for 25 years, and in one case the 5-trials were incomplete and no k-value could be calculated
